# Antimicrobial and Antioxidant Efficacy of the Lipophilic Extract of *Cirsium vulgare*

**DOI:** 10.3390/molecules28207177

**Published:** 2023-10-19

**Authors:** Mine Aydın Kurç, Hakime Hülya Orak, Dumrul Gülen, Hilmican Caliskan, Merve Argon, Temine Sabudak

**Affiliations:** 1 Department of Medical Microbiology, Faculty of Medicine, Tekirdag Namik Kemal University, 59030 Tekirdag, Turkey; dgulen@nku.edu.tr; 2 Department of Food Technology, Vocational School of Technical Sciences, Tekirdag Namik Kemal University, 59030 Tekirdag, Turkey; horak@nku.edu.tr; 3 Department of Chemistry, Faculty of Science and Arts, Tekirdag Namik Kemal University, 59030 Tekirdag, Turkey; hlmcn.clskn@gmail.com (H.C.); merweozer92@gmail.com (M.A.); tsabudak@nku.edu.tr (T.S.)

**Keywords:** *C. vulgare*, GC–MS analyses, semi-volatile compound, antifungal, antibacterial, antioxidant activity

## Abstract

The aim of this study was to investigate the compounds in the hexane extract of *Cirsium vulgare* (Savi.) Ten. and to determine the antibacterial, antifungal, and antioxidant activities of different extracts. The *Cirsium vulgare* (NGBB 7229) plant was collected from Turkey’s Trakya region. Crude extracts were obtained using different solvents. The chemical composition of *Cirsium vulgare* was determined in hexane extract using gas chromatography mass spectrometry. The antioxidant activities of the extracts were evaluated by Trolox equivalent antioxidant activity (TEAC), ferric-reducing antioxidant power (FRAP), cupric-reducing antioxidant capacity (CUPRAC), the β-carotene bleaching method, and the determination of superoxide anion scavenging activities. The antibacterial activity was tested against *Staphylococcus aureus*, *Bacillus subtilis*, *Escherichia coli*, *Pseudomonas aeruginosa*, *Proteus mirabilis*, and *Salmonella typhimurium*, whereas the antifungal activity was tested against *Candida albicans, Candida glabrata, Candida parapsilosis*, *Candida krusei*, *Penicillium chrysogenum,* and *Aspergillus fumigatus* by applying microdilution methods. A total of 41 bioactive compounds were identified using the GC–MS library. Terpenoids were found to be dominant (52.89%), and lup-20(29)-en-3-yl-acetate and lupeol were the most abundant terpenoids. The highest total flavonoid content (25.73 mg catechin/g) and antioxidant capacity were found in the methanolic extract. The highest antibacterial activity was detected against *Bacillus subtilis* in the ethyl acetate extract, and the highest antifungal activity was found against *Candida krusei* and *Aspergillus fumigatus* in the hexane extract. The observed antioxidant characteristics of the *C. vulgare* extracts could be attributed to the presence of flavonoids. The high antifungal activity of the hexane extract against all fungal strains can be attributed to its constituents, i.e., terpenoids. This study discloses the potential antioxidant and antimicrobial activities, including some bioactive components, of *Cirsium vulgare* and implies that *Cirsium vulgare* holds possible applications in the food and pharmaceutical industries as an antioxidant, antibacterial, and antifungal agent.

## 1. Introduction

Bioactive compounds obtained from different sources exhibit great potential for preventing free radical damage when used as functional food components and antioxidant agents in the food or pharmaceutical industries. On the other hand, safer alternative bactericides and fungicides are needed to reduce resistance to synthetic antimicrobials. Therefore, drugs derived from natural sources play a significant role in the prevention and treatment of human diseases.

The *Asteraceae* family is one of the largest families of flowering plants, with about 1600 genera and over 23,000 species; the bioactivities of a number of the *Asteraceae* species have not yet been investigated [1]. The *Cirsium* genus belongs to the *Asteraceae* family and is believed to be harmful in agricultural areas, as it can exhibit uncontrollable reproduction and growth. But the stem and roots of different *Cirsium* genus have been used as a food source and food additive in rural areas in Turkey for years [2]. In different countries, different species of *Cirsium* have been used in hepatoprotective folk medicine [2,3,4]. Some species have been used traditionally for the treatment of gastritis, diabetes, hemorrhoids, and cough [5]. The leaves and stems of many species are also edible and can be used in tea, soup, and salads [6,7]. There are several reports regarding the antioxidant, antimicrobial [2,8,9,10], antidiabetic [11], and anti-tumor activities [12] of some *Cirsium* species. More than ten species of *Cirsium* have been used as folk medicines, and modern pharmacological studies have shown that *Cirsium* exhibits liver protection, along with antioxidant, anti-tumorigenesis, anti-inflammation, antibacterial, and other beneficial effects [13].

*C. vulgare* is present in a wide variety of habitats, mostly with a high degree of disturbance [14]. Our recent study revealed that the extracts from *C. vulgare* showed DPPH radical scavenging activity and antibacterial activity, according to zone diameters [15]. Previous phytochemical studies have reported that *C. vulgare* contains flavonoids and phenolic compounds [16,17]. In addition to these polar compounds, non-polar compounds, such as terpenes and fatty acids, have been discovered from *Cirsium* species [18]. In a recent study, Fernández-Martínez et al. [19] suggested that despite their non-polar constituents, the hexane extracts are not free radical scavengers, as is the case for the flavonoids of the *Cirsium* polar extracts.

Recently, attention has been focused on natural plant products, used alone or in combination with synthetic fungicides, for use in the food and pharmaceutical industries. Moreover, recent literature screened the most promising examples of dual-active antimicrobial–antioxidant sources, and *C. vulgare* was determined to be promising. The phytochemical studies of different *Cirsium* species and their renowned pharmacological activities could be exploited for pharmaceutic product development [20]. Shahrajabian [21] reported that spear thistle (*C. vulgare*) can promote good health and serve as a primary defense mechanism against diseases. The aim of the present study was to analyze the composition of volatile/semi-volatile compounds of hexane extract and to determine the total flavonoid content; the antibacterial, antifungal, and antioxidant efficacy of lipophilic extracts (hexane and diethyl ether); and the ethyl acetate and methanol extracts obtained using different extraction methods of wild *C. vulgare*. There is no study in the current literature regarding the composition of hexane extracts of *C. vulgare.* The present work is the first report on the antibacterial and antifungal activities of *C. vulgare* in terms of minimum inhibitory concentration (MIC) values and various antioxidant properties of different extracts.

## 2. Results and Discussion

### 2.1. GC–MS Analysis

The GC–MS chromatogram of hexane extract revealed the presence of 41 compounds. These compounds were characterized by their retention time (RT), retention index (RI), and their molecular formula and concentrations, according to the peak area (%), and are presented in Table 1. In this study, GC–MS results indicated that the hexane extract is a rich source of terpenoid compounds, and terpenoids exhibit antimicrobial, antiviral, antiallergic, and anti-inflammatory activities [22]. According to chemical class distribution, terpenoids (52.89%) were the most abundant compound, followed by esters (19.92%) and hydrocarbons (14.17%) (See Table 2). In the determination of the volatile components of the *C. japonicum* plant using GC–MS, Miyazawa et al. found terpenoids (45.22%) to be the most abundant compounds [23]. Lup-20(29)-en-3-yl-acetate (29.94%), lupeol (13.19%), linolenic acid ethyl ester (6.38%), norolean-12-en (5.15%), 1-nonadecene (4.23%), 9,12-octadecadien-1-ol (4.9%), hexadecanoic acid (palmitic acid) (3.21%), 1-tricosene (2.89%), and cycloeicosane (2.62%) were the major compounds found in the extract (Table 1). In the study conducted by Orhan et al. [24], palmitic acid was observed as the main component in hexane extract of *C. hypoleucum*. Additionally, Kozyra et al. [6] detected β-linalool (1.3%), β-cyclocitral (1.7%), and eugenol (1.7%) terpenoids in the essential oil analysis of the *C. vulgare* plant using the GC–MS method.

The most abundant terpenoids in the hexane extract were Lup-20(29)-en-3-yl-acetate, lupeol, norolean-12-ene, and methyl commate B (2.0%). Lupeol and lup-20(29)-en-3-yl-acetate are triterpenes that exhibit pharmacological activities, including anticancer, anti-inflammatory, and antimicrobial properties [25]. Lupeol also shows nephroprotective and hepatoprotective effects [26]. Methyl commate B is a pentacyclictriterpene glycoside which possesses antimicrobial and anti-inflammatory properties [27]. Hydrocarbons were also (14.17%) quite abundant, with significant amounts of 1-nonadecene (4.23%), 1-tricosene (2.89%), and cycloeicosane (2.62%). The n-alkanes are thought to be indigenous to plants and are formed as the result of the decarboxylation of long-chain fatty acids [28]. The long aliphatic hydrocarbons are found on the surfaces of the aerial organs of plants and are important for repelling water and controlling the gas balance within a plant [29]. Smaoui et al. [30] reported that 1-nonadecene from the *Streptomyces* sp. TN 256 strain exhibited antibacterial activity and strong antifungal activity against *C. albicans* [31]. Among the esters, hexadecanoic acid butyl ester (butyl palmitate) (9.89%) and linolenic acid ethyl ester (6.38%) were predominant. 9,12-Octadecadien-1-ol (linoleyl alcohol) (4.9%), which is a fatty alcohol produced by the reduction of linolenic acid, and palmitic acid (4.9%) were the major compounds found in the hexane extract. Leventhal et al. [32] reported that fatty acids can modulate immune responses, and Reifen et al. [33], suggested that α-linolenic acid has potential as an anti-inflammatory agent. A similar explanation was provided by Aparna et al., who reported that hexadecanoic acid may be an anti-inflammatory compound [34]. According to the literature; β-linalool (1.3%), β-cyclocitral (1.7%) and eugenole (1.7%) values were also revealed in the GC–MS analysis of *C. vulgare* plants [6]. Additionally, Orhan et al. [24] suggested that palmitic acid (26.35%) is the main fatty acid identified in the *C. hypoleucum*.

### 2.2. Total Flavonoids Content

The highest TFC was found in methanol extract, determined as 25.7 mg catechin and 44.6 mg RE in the gram extract (Table 3), while the highest total flavonoid content was found in the methanol extract, followed by ethyl acetate and then diethyl ether extract. TFC was not found in the hexane extract. Our findings are lower than those obtained by Nazaruk et al., who determined a TFC between 170–209 mg catechin equivalent in one g extract of *C. vulgare* [35]. According to antioxidant activity assays, methanol extract showed the highest antioxidant activity in all tested antioxidant assays. Recently reported by Griškevičienė et al. [36], the highest amounts of flavonoids obtained by heating with reflux from *C. vulgare* leaves were rutin, hyperoside, isoquercitrin, chlorogenic acid, and apigenin-7-*O*-glucoside, respectively.

### 2.3. Antioxidant Activity

The highest TEAC capacity was determined in methanol extract (0.86 mmol Trolox/g). The hexane fraction exhibited the lowest activity, with a 0.34 Trolox/g value. According to the results, the methanol extract exhibited 2.53 times more activity than did the hexane extracts (Table 3). The ABTS^•+^ radical reducing ability results of the *C. vulgare* extracts are in agreement with the findings of Malejko et al., in which they determined 3.23 times higher ABTS activity in the refluxed methanolic extracts than in ethyl acetate for the *C. palustre* extracts (Table 3) [37]. Zhao et al. [38] found that the various parts of seven *Cirsium* species in Taiwan showed varying degrees of antioxidant activities against free radicals in regards to the 16 methanolic standards, according to the ABTS and DPPH methods.

The highest FRAP capacity was determined for methanol extract (1436.6 µmol Fe^2+/^g extract), and the lowest value was obtained for hexane extract (49.7 µmol Fe^2+/^g extract). Based on the EC_50_ value of the FRAP assay, the strength of FRAP power was in the order of: methanol (14.7 µg/mL) > ethyl acetate (80.5 µg/mL) > diethyl ether (127.5 µg/mL) > hexane (651.8 µg/mL). The differences between FRAP activities in different solvent extractions could be explained by solvent polarity, as the use of different solvents of varying polarities may lead to higher and lower mass transfers of different plant phenolics.

The CUPRAC assay is a redox potential-based method, and the results of the CUPRAC assay determined that the highest activity is obtained from methanol extract (2.14 mmol Trolox/g; EC_50_ of 18.5 µg/mL) and ethyl acetate extract, followed by values of 1.62 mmol Trolox/g and EC_50_ of 33.5 µg/mL. The hexane fraction exhibited the lowest activity (0.38 mmol Trolox/g extract; EC_50_of 140.6 µg/mL) (Table 3). It was shown here that the potency of methanol extract was around 1.81, 3.27, and 7.6 times as high as the potency of the ethyl acetate, diethyl ether, and hexane extracts, respectively. The values are higher than those reported by Karasakal et al., who reported lower CUPRAC values (0.18 mmol/g) after 80% methanol extraction in the *C. vulgare* varieties [39]. On the contrary, Boga et al. reported that acetone and methanol extracts and isolated compounds from two endemic *Cirsium* species and *C. eriophorum* grown in Turkey [40] did not show CUPRAC activity. Higher CUPRAC activities in the methanol, ethyl acetate, and diethyl ether extracts compared to those of the hexane extract could be explained by differences in solvent polarity, similar to the results found for the TEAC and FRAP capacities of the previously mentioned extracts.

**Figure 1:**
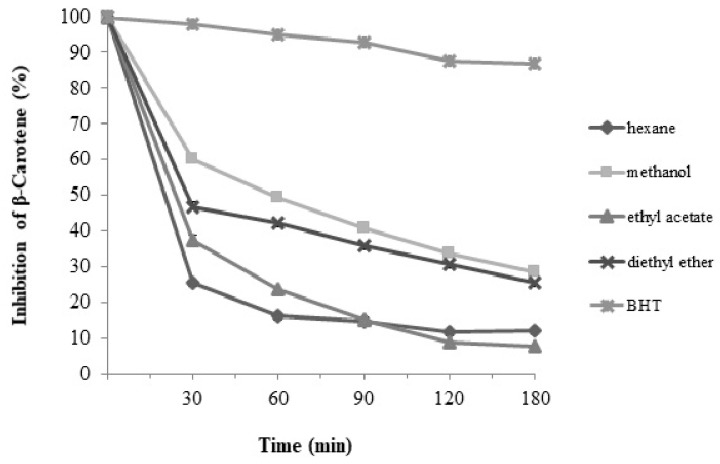
Inhibition of the oxidation of β-carotene-linoleic acid emulsion by *C. vulgare* extracts; BHT butyhlhidroxytoluene; (*n* = 3).

In the β-carotene-linoleic acid emulsion model, the hexane extracts showed the lowest inhibition effect. The highest efficient antioxidant activity in the lipid system seems to be related to the compounds extracted by methanol, similar to the ABTS and FRAP activity results. According to the results, 28.47% of β-carotene in the methanol extracts remained non-oxidized at the end of the oxidation reaction time (180 min). The inhibition of β-carotene was determined as 25.32% for diethyl ether, 12.13% for hexane, and 7.72% for ethyl acetate extract. According to the results, methanol extract presented the highest flavonoid content and the highest effect against the oxidation of β-carotene in the linoleic acid emulsion system (Figure 1). Nazaruk et al. [35] found that ethyl acetate extract from the *C. vulgare* flower exerted a 38.5% inhibition effect on β-carotene after one hour, and in this study, we determined higher inhibition effects for methanol and diethyl ether extracts after one hour (49.33% and 42.11%, respectively). However, different extraction methods were used.

The superoxide anion radical scavenging activity of the extracts at a concentration of 1 mg/mL are given in Table 3, and the results are compared to those for l-ascorbic acid. According to our results, methanol extract exhibited strong superoxide radical scavenging activity comparable to that of l-ascorbic acid. The inhibition of superoxide anion was found to be 74.85%, whereas that of l-ascorbic acid was found to be 99.08%. Demirtas et al. also reported the occurrence of higher superoxide anion radical scavenging activity in the *C. arvense* methanol-chloroform extracts compared to that of standard compounds, namely a-tocopherol, BHT, and BHA [2].

### 2.4. Antibacterial Activity

The antibacterial MIC levels of *C. vulgare* extracts against *S. aureus* were in the range of 15.62–250 mg/mL (Table 4). Diethyl ether and ethyl acetate extracts exhibited the highest inhibition effect on *S. aureus*, with MIC values of 15.62 mg/mL. All extracts had an effect on *B. subtilis*, and the MIC values ranged from 3.9 to 250 mg/mL. The highest inhibition effect was found in ethyl acetate extract, with an MIC level of 3.9 mg/mL. The MIC levels of four different extracts for *E. coli* were in the range of 15.62–125 mg/mL. The hexane extract showed highest inhibition effect on *E. coli.* The MIC levels of extracts for *P. aeruginosa* and *P. mirabilis* were in the ranges of 15.62–250 mg/mL and 31.25–250 mg/mL, respectively, and the diethyl ether extract exhibited the highest inhibition effect. The MIC levels of extracts for *S. typhimurium* were in the range of 31.25–250 mg/mL, and the diethyl ether extract showed the highest inhibition effect. According to antimicrobial activity results, the current study revealed that the highest antibacterial activity was found against *B. subtilis* in the ethyl acetate extract. Kenny et al. reported that neither the ethanol nor water extracts generated from *C. arvense* and *C. vulgare* exhibited any activity against *S. aureus*, MRSA, *B. cereus*, *E. coli,* or *S. typhimirium* [1]. Conversely, the water and ethanol extracts of *C. palustre* were active against *S. aureus*, while the ethanol extract showed further inhibition against strains of MRSA (MIC = 375 µg/mL), *B. cereus* (MIC = 500 µg/mL), and *E. coli* (MIC = 375 µg/mL). A study by Karasakal et al. indicated that *C. bulgaricum* demonstrated antimicrobial activity against a range of bacteria, including *E. coli* (MIC values = 250 and 500 µg/mL), *S. enteritidis* (MIC = 500 µg/mL), *L. monocytogenes* (MIC = 250 µg/mL), and *S. aureus* (MIC = 250 µg/mL) [39]. Borawska et al. also confirmed the antimicrobial activity of flower and leaf extracts from *C. arvense*, *C. oleraceum*, *C. palustre*, *C. rivulare,* or *C. vulgare* against *S. aureus*, *B. subtilis,* and *P. aeruginosa* [41]. Loizzo et al. observed that the herbal acetate extract from *C. tenoreanum* inhibited the growth of *S. aureus* (MIC = 0.5 mg/mL) and *E. coli* (MIC = 1 mg/mL) [42]. Nazaruk and Jakoniuk proved that aqueous, methanol, and 70% ethanolic extracts from *C. rivulare* flowers and leaves also showed some antimicrobial activity, in which the aqueous leaf extract exhibited high activity, especially against Gram-positive bacteria. These extracts demonstrated antimicrobial activity against *S. aureus* (MIC = 6.2–25 mg/mL), *B. subtilis* (MIC = 6.2–25 mg/mL), *E. coli* (MIC = 6.2–50 mg/mL), and *P. aeruginosa* (MIC = 6.2–50 mg/mL) [43]. Kozyra et al. reported that extracts from *C. canum* had no influence on the growth of the reference strains of Gram-negative bacteria and of yeasts belonging to *Candida* spp. However, the fractions possessed the highest activity against Gram-positive bacteria, especially *S. aureus* (MIC = 125–1000 µg/mL) and *S. pneumonia* (MIC = 125–1000 µg/mL), which are pathogens; and *S. epidermidis* (MIC = 125–1000 µg/mL), *B. cereus* (MIC = 62.5–1000 µg/mL), and *B. subtilis* (MIC = 125–1000 µg/mL) which are opportunistic microorganisms [18]. Kozyra et al. isolated essential oils from the herb of *C. vulgare*, proving antimicrobial activity for Gram-positive and Gram-negative bacteria (concentration: 20 mg/mL) [6]. A study conducted by Gadisa and Tadesse [44] showed that methanol extract of *C. englerianum* showed antibacterial activity against *S. aureus* (MIC = 16 µg/mL), *E. faecalis* (MIC = 1 µg/mL), *E. coli* (MIC = 64 µg/mL), and *K. pneumoniae* (MIC = 2 µg/mL). Another study using *C. englerianum* extract showed that the plant possessed inhibitory potential in regards to multidrug-resistant and the reference strains. This methanol extract demonstrated inhibitory activity against *S. aureus* (MRSA and MSSA) (MIC = 16 µg/mL), *S. pyogenes* (MIC = 1 µg/mL), *E. coli* (MIC = 64 µg/mL), and *K. pneumoniae* (MIC = 2 µg/mL) [45]. Shahid et al. [46] reported that methanol extract from *C. swaticum* Petr. showed antimicrobial activity against *S. aureus*, *S. typhi*, *B. megaterium*, *B. subtilis*, *P. mirabilis,* and *E. coli*. In this study, extracts from *C. vulgare* possessed the highest antibacterial activity, especially against *B. subtilis*, *S. aureus*, *E. coli,* and *P. aeruginosa*.

### 2.5. Antifungal Activity

Antifungal MIC levels of extracts against *C. albicans* were in the range of 1.95–250 mg/mL (Table 5), and the highest inhibition effect was determined in the hexane extract. The MIC levels against *C. glabrata*, *C. parapsilosis*, and *C. krusei* were in the ranges of 1.95–125 mg/mL, 1.95–15.62 mg/mL, and 0.97–31.25 mg/mL, respectively, and the highest inhibition effect was determined for the hexane extract. The MIC levels of four different extracts against *P. chrysogenum* and *A. fumigatus* were in the ranges of 3.9–31.25 mg/mL and 0.97–250 mg/mL, respectively. Similarly, the highest inhibition effects were found in the hexane extract (Table 5).

To the best of our knowledge, the present work is the first report on the antibacterial and antifungal activities of *C. vulgare* in different extracts in terms of MIC values and various antioxidant properties. According to our study results, the highest antifungal activity was found against *C. krusei* and *A. fumigatus* in the hexane extract. Current information regarding the antifungal efficacy of extracts from the *Cirsium* species is scarce. In some studies [6,18], no antifungal activity was detected against fungal strains of extracts from the *Cirsium* species. A study by Nazaruk and Jakoniuk proved that aqueous, methanol, and 70% ethanol extracts from *C. rivulare* flowers and leaves showed inhibitory activity against *C. albicans* (MIC = 25–50 mg/mL) [43]. Ozcelik et al. reported that various extracts from *C. hypoleucum* showed antifungal activity against *C. albicans* (MIC = 64 µg/mL) and *C. parapsilosis* (MIC = 64 µg/mL) [47]. In other studies, methanol extracts of *C. englerianum* showed antifungal effects on *C. albicans*, with MIC values of 128 µg/mL [43,44]. In our study, hexane extract exhibited the highest inhibition effects against all tested fungal strains. However, no previous literature study reported the antifungal activity of the extracts of *C. vulgare*, as demonstrated in this study.

## 3. Materials and Methods

### 3.1. Plant Material

In this study, the whole plant parts of *C. vulgare* (root, stem, leaf, and flower) were collected from a natural habitat in the Trakya region of Turkey in June 2016. These plants were identified by Prof. Dr. E. Cabi of the Faculty of Science, Department of Biology, at Tekirdag Namik Kemal University, and a voucher specimen was deposited with the voucher number NGBB 7229.

### 3.2. Extractions

The whole plant parts (228 g) were ground and homogenized after being dried at room temperature. The extractions were carried out for 3 days, and a total of two macerations were performed in each solvent. The ground plants were macerated at room temperature using pure hexane (5.819 g), diethyl ether (0.973 g), ethyl acetate (0.905 g), and methanol (4.228 g) as solvents. The solvents were evaporated under vacuum using a rotary evaporator (Büchi Labortechnik, Flawil, Switzerland, Model: R-210 Rotavapor). The extraction yields were calculated as 2.55% for hexane extract, 0.42% for diethyl ether extract, 0.39% for ethyl acetate extract, and 1.58% for methanol extract. Then, the compositions of volatile compounds for the hexane extract of *C. vulgare* were investigated using GC, and the antibacterial, antifungal, and antioxidant activities were determined for all four extracts.

### 3.3. GC–MS Analysis

Chromatographic analyses were conducted using a Hewlett-Packard HP 6890 series GC/MS device. HP-5MS (5% phenyl methyl siloxane, 30 m × 250 μm × 0.25 μm) was used as the capillary column. Helium was used as the carrier gas, at a flow rate of 1.0 mL/min. The column’s initial temperature was 180 °C 1 min after injection. The temperature was increased to 250 °C with an 8 °C/min heating ramp and a 1 min hold time, and the temperature was increased to 300 °C, with a 2 °C/min heating ramp over 10 min. The injection was performed in split mode (split ratio: 10:1). For analysis, the interface temperature was 250 °C, the injector temperature was 280 °C, and the running time was 49 min. The MS scan range was *m*/*z* 20–1000 using electron impact (EI) ionization (70 eV) and an ion source temperature of 250 °C. The components were identified by the comparison of their mass spectra with those of Wiley 9 and the NIST library. The relative percentages of the separated compounds were calculated with total ion chromatography using the computerized integrator. The retention indices (RI) were recognized externally using a series of n-alkanes (C6–C22), under the same chromatographic conditions [24].

### 3.4. Total Flavonoids Content (TFC)

Total flavonoid content was determined according to the suggestions of Zhishen et al. [48] by using the AlCl_3_-NaNO_2_ method at a wavelength of 510 nm. A 0.25 mL aliquot of extract was mixed with 1.25 mL of distilled water in a test tube, followed by the addition of 75 μL of 5% sodium nitrite solution. After an incubation time of 6 min, 150 µL of 10% aluminium chloride was added. After 5 min, 0.5 mL of 1 M sodium hydroxide solution was added to the mixture. The mixture was immediately diluted to 2.5 mL by adding distilled water, mixing thoroughly. The absorbance of the mixture, which is pink in color, was determined at 510 nm against a blank containing all reagents except the extract samples. The total flavonoid content of the *C. vulgare* was calculated as mg catechin (CAT) and rutin (RE) equivalents per gram of the extracts (mg/g). The total flavonoid content was calculated with the help of the the standard curve equation: y = 21,782x + 0.0349, where R^2^ = 0.9938 for catechin, and y = 12,714x + 0.0017, where R^2^ = 0.9941 for rutin.

### 3.5. Antioxidant Activities

#### 3.5.1. Trolox Equivalent Antioxidant Activity (TEAC)

The Trolox equivalent antioxidant capacity (TEAC) was estimated by using the method of Re et al. [49]. For this assay, 2,2′-azino-bis(3-ethylbenzothiazoline-6-sulfonic acid) cation radical (ABTS^•+^) solution was prepared by dissolving 96 mg of ABTS in 2.45 mmol/L Na_2_S_2_O_8_. This solution was shaken for 16 h at room temperature in the dark until a stable oxidative state was achieved. The ABTS^•+^ stock solution was diluted with methanol to an absorbance of 0.70 ± 0.02 at 734 nm before analysis. For the spectrophotometric assay, 2 mL of the ABTS^•+^ solution and 20 μL of *C. vulgare* extracts were mixed, and the absorbance was recorded at 734 nm (Hitachi spectrophotometer, 121-002.IR) after incubating the samples at 30 °C for 6 min. The calibration curve was plotted by using 6-hydroxy-2,5,7,8-tetramethylchromane-2-carboxylic acid (Trolox) as a standard. The results were expressed as mmol Trolox equivalents per g of extract, with the help of the the standard curve equation, which was y = 50,152 Trolox (mmol) + 93,842 (R^2^: 0.993).

#### 3.5.2. Ferric-Reducing Antioxidant Power

The ferric-reducing antioxidant power (FRAP) assay was performed as previously described by Benzie and Strain [50]. The working FRAP reagent was prepared by mixing 10 volumes of 300 mmol/L acetate buffer, pH 3.6, with 1 volume of 10 mmol/L 2,4,6-tris(2-pyridyl)-S-triazine (TPTZ) in 40 mol/L HCl and with 1 volume of 20 mmol/L FeCl_3_ × 6H_2_O. A volume of 2.25 mL of a working FRAP reagent was warmed to 37 °C. Then, 75 μL of the sample and 225 μL of deionized water were added to the FRAP reagent, and the absorbance was measured at 593 nm against reagent blank after 30 min of incubation. The results were expressed as μmol Fe^2+^ equivalents per g of extract with the help of the calibration curve prepared in the concentration range of 0.1–1.0 μmol/mL FeSO_4_ × 7H_2_O (y = 0.6655 Fe^2+^ (μmol) + 0.0021 (R^2^: 0.9978). The extract concentration providing 0.5 absorbance (EC_50_) was calculated from the graph of absorbance at 593 nm against the extract concentration range of 0.0292–0.0882 mg/mL.

#### 3.5.3. β-Carotene-Linoleic Acid Emulsion Oxidation

The β-carotene bleaching test in the β-carotene-linoleic acid emulsion system was determined according to the methods suggested by Miller [51]. A total of 1.6 mg of β-carotene was dissolved in 2 mL of chloroform, and then 400 mg of Tween 40 and 40 μL of linoleic acid were added to prepare the emulsion. The chloroform was evaporated, and 2 mL of methanol and 50 mL of water were added to the residue. A total of 250 µL of the emulsion mixture was vortexed with 10 μL of the extract solution (1 mg/mL). Methanol was added to the control sample. The absorbance of the samples was measured at 470 nm at 30 min intervals throughout the 180 min oxidation process. The reaction temperature was 42 °C. The results were expressed as the percentage of non-oxidized β-carotene after 180 min of reaction. The absorbance of the extracts and the control were measured immediately (t = 0). The tubes were incubated at 42 °C, and the absorbance was measured using a spectrophotometer at 30 min intervals at 470 nm for 180 min (t = 180). The antioxidant activity (AA) of the extracts was evaluated in terms of bleaching of the β-carotene using the following formula:

AA = [(1 − (A_0_ − A_t_/A_0_^0^ − A_t_^0^)] × 100

where A_0_—absorbance value of the extract at zero time of incubation; A_t_—absorbance value of the extract at t minutes of incubation; A_0_^0^—absorbance value of the control at zero time of incubation; A_t_^0^—absorbance value of control at t minutes of incubation; BHT (1 mg mL^−1^) was taken as the positive control sample.

#### 3.5.4. Cupric-Reducing Antioxidant Capacity (CUPRAC)

For determination of the CUPRAC activity, CuCl_2_ solution (1.0 × 10^−2^ M), neocuproine alcoholic solution (7.5 × 10^−3^ M) and NH_4_Ac buffer solution (pH = 7) were used for the analyses [52], and absorbance readings were obtained at 450 nm. The CUPRAC activity of the extracts (mM trolox/g) was calculated from the calibration curve obtained using Trolox as standard. The extract concentration providing 0.5 absorbance (EC_50_) was calculated from the graph of absorbance at 450 nm against the µg/mL extract concentration.

#### 3.5.5. Superoxide-Radical Scavenging Activity

Superoxide anion scavenging activities were determined according to the method described previously by Robak and Gryglewski [53]. The reaction mixtures were arranged in 0.1 M phosphate buffer at pH 7.4. A total of 1 mL (156 μM) of nitrobluetetrazolium (NBT), 1 mL (468 μM) of reduced nicotinamide adenine dinucleotide (NADH), and 1 mL of the extracts were mixed at 100 μg/mL concentrations. A total of 100 μL of phenazine methosulphate (PMS, 60 μM) was added for reaction initiation. Incubation was performed at 25 °C for 5 min. Absorbance was measured at 560 nm using l-ascorbic acid as a control. The percentage of inhibition was determined using the following formula:

superoxide inhibition percentage = [(A_0_ − A_1_)/A_0_] × 100

where A_0_—absorbance of the control, and A_1_—absorbance of the extracts.

### 3.6. Antimicrobial Activities

#### 3.6.1. Microorganisms

The antibacterial activity was tested against two Gram-positive (*Staphylococcus aureus* ATCC 43300 and *Bacillus subtilis* NRRL NRS-744) and four Gram-negative bacterial strains (*Escherichia coli* ATCC 35218, *Pseudomonas aeruginosa* ATCC 27853, *Proteus mirabilis* ATCC 12453, and *Salmonella typhimurium* ATCC 14028) that were grown in nutrient agar at 37 °C for 18 h. The antifungal activity was tested against six fungal strains (*Candida albicans* ATCC 90028, *Candida glabrata* ATCC 90030, *Candida parapsilosis* ATCC 22019, *Candida krusei* ATCC 6258, *Penicillium chrysogenum* ATCC 48271, and *Aspergillus fumigatus* ATCC 204305) that were grown in Sabouraud dextrose agar (SDA) at 27 °C.

#### 3.6.2. Minimum Inhibitory Concentration (MIC)

The plant extracts were dissolved in H_2_O and DMSO at 1000 mg/mL of stock concentration to determine their antibacterial and antifungal activities. Penicillin G, gentamycin, and fluconazole were used as the standard antibacterial and antifungal drugs. These tests were conducted by applying the microdilution method in a liquid medium, according to CLSI standards [54,55,56]. The procedure involves preparing two-fold dilutions of the plant extracts (e.g., 0.48, 0.97, 1.95, 3.90, 7.81, 15.62, 31.25, 62.5, 125, and 250 mg/mL) in a liquid growth medium (Mueller-Hinton Broth and RPMI 1640). The MIC values were determined as the lowest concentration of extracts inhibiting the visible growth of each organism on the plate.

## 4. Conclusions

In addition to the determination of some phytocompounds in the hexane extract, this study reports the antioxidant, antibacterial, and antifungal activity of different organic solvent extracts of *C. vulgare*. The highest antibacterial activity was found against *B. subtilis* in the ethyl acetate extract, while the highest antifungal activity was found against *C. krusei* and *A. fumigatus* in the hexane extract. According to the results of the antioxidant assays, the highest observed antioxidant activity of the *C. vulgare* extracts in the methanol extract could be attributed to the presence of extractable flavonoid compounds and a high flavonoid content. The highest levels of total flavonoids were found in the polar methanol extract, followed by the ethyl acetate because the flavonoid class compounds tend to be semipolar–polar, so that more flavonoid compounds could be extractable in semipolar–polar solvents, such as methanol, ethyl acetate, and diethyl ether, compared to that obtained from hexane. *C. vulgare* may provide even higher antioxidant activities to the methanol extracts than to the hexane extract.

On the other hand, the hexane extract exhibited a high antifungal activity against all fungal strains; therefore, we can conclude that constituents such as terpenoids, esters, and hydrocarbons found in lipophilic extract could be responsible of their high bioactivity and the reinforcement of these actions. The GC–MS analysis of the hexane extract of *C. vulgare* showed the existence of some important chemical compounds with different chemical structures. The present work discloses the potential antioxidant and antimicrobial activities of *C. vulgare*, along with some bioactive components, indicating that *C. vulgare* might hold potential for use as an antioxidant, antibacterial, and antifungal agent in food and pharmaceutical industries.

## Figures and Tables

**Table 1:** The composition (%) of volatile compounds of the hexane extract of *C. vulgare*.

No.	RI	Compounds	Percentage [%]
1	891	5-Eicosene	1.11
2	891	Cycloeicosane	2.62
3	936	Hexadecanoic acid (palmitic acid)	3.21
4	941	1-Nonadecene	4.23
5	975	Hexadecadienoic acid, methyl ester	0.74
6	986	9,12-Octadecadien-1-ol	4.90
7	994	*cis*-9-Hexadecenal	1.15
8	1002	Hexadecanoic acid, butyl ester	9.89
9	1043	Docosane	0.50
10	1069	Linolenic acid, ethyl ester	6.38
11	1080	1-Tricosene	2.89
12	1129	Pentacosane	0.41
13	1156	1,2-Benzenedicarboxylic acid, diisooctyl ester	0.36
14	1165	Tetrahydrogeraniol	0.17
15	1183	Pentafluoropropionic acid, heptadecyl ester	1.99
16	1228	17-Pentatriacontene	0.18
17	1263	10-Methylnonadecane	0.24
18	1273	1,3-Benzenedicarboxylic acid, bis(2-ethylhexyl) ester	0.33
19	1292	*cis*-9,10-Epoxyoctadecanamide	0.31
20	1330	Squalene	0.11
21	1407	2,2-dimehyl-3-(3,7,16,20-tetramethyl-heneicosa-3,7,11,15,19-pentaenyl) oxirane	0.15
22	1457	1-Hentetracontanol	0.51
23	1470	Acetic acid, octadecyl ester	0.11
24	1492	Octadecanal	0.13
25	1590	Solanesol	0.14
26	1548	Tetratetracontane	1.99
27	1572	Vitamin E	0.16
28	1583	1,10-dibromo-decane	0.10
29	1648	1-Triacontanol	0.27
30	1682	Octadecanal	0.09
31	1684	Stigmasterol	0.35
32	1740	Stigmast-5-en-3-ol	1.17
33	1774	Olean-12-ene	1.82
34	1786	1,54-dibromo-tetrapentacontane	0.19
35	1813	Methyl commate B	2.00
36	1858	Docosyl acetate	0.12
37	1860	A′-Neogammacer-22(29)-ene (Diploptene)	0.37
38	1882	Norolean-12-ene	5.15
39	1936	Lupeol	13.19
40	2066	Lup-20(29)-en-3-yl-acetate	29.94
41	2301	4′,5-dihydroxy-7-diglyocoside-flavone	0.31

**Table 2:** The chemical class distribution of the *C. vulgare* hexane extract.

Volatile Compounds	Percentage [%]
Terpenoids	52.89
Hydrocarbons (alkanes and alkenes)	14.17
Esters	19.92
Alcohols	5.68
Fatty acids	3.21
Sterols	1.52
Carbonyl compounds (aldehyde-ketones)	1.37
Other functional groups (amide, epoxide, alkyl halide, etc.)	0.29 alkyl halide 0.31 amide 0.31 phenolics 0.15 epoxide 0.16 vitamin E
Total (%)	99.98

**Table 3:** The total flavonoid content (TFC) and antioxidant activities of *C. vulgare* extracts.

Extracts	^1^ Total Flavonoid Content		Antioxidant Activity Assays					
	TFC (mg CAT/g)	TFC (mg RUT/g)	TEAC (mmol Trolox/g)	FRAP (μmol Fe^2+^/g)	^2^ EC_50_ FRAP (µg/mL)	CUPRAC (mM Troloks/g)	^3^ EC_50_CUPRAC (µg/mL)	^4^ Superoxide-Radical Scavenging Activity (%)
Methanol	25.7 ± 2.2	44.6 ± 1.3	0.86 ± 0.04	1436.6 ± 11.1	14.7 ± 0.08	2.14 ± 0.002	18.5 ± 0.002	74.8 ± 0.8
Ethylacetate	23.6 ± 0.9	40.3 ± 1.8	0.41 ± 0.01	295.6 ± 4.1	80.5 ± 0.02	1.62 ± 0.002	33.5 ± 0.031	53.5 ± 1.2
Hexane	nd ^5^	nd	0.34 ± 0.03	49.7 ± 3.7	651.8 ± 0.01	0.38 ± 0.055	140.6 ± 0.003	24.9 ± 0.2
Diethyl ether	16.3 ± 0.8	28.1 ± 0.8	0.40 ± 0.02	115.3 ± 4.7	127.5± 0.03	1.15 ± 0.011	60.6 ± 0.001	39.8 ± 2.5

^1^ The total flavonoid content was calculated as catechin and rutin equivalents in g extract, where the catechin equivalent was obtained according to y = 21.782 (CAT) + 0.0349 (R^2^: 0.9988), and the rutin equivalent was obtained according to the y = 12.714 (RUT) + 0.0017 (R^2^: 0.9941) equation. ^2,3^ The EC_50_ value (μg/mL) is the effective concentration giving an absorbance of 0.5, which indicates the concentration of the extract that yields a half-maximal response (efficient concentration = EC_50_). ^4^ ascorbic acid exhibited 99.09 ± 1.05% activity at the same concentration; ^5^ not detected.

**Table 4:** Antibacterial MIC results (mg/mL) for different extracts.

Bacteria	Methanol	Ethyl Acetate	Diethyl Ether	Hexane	Standard (µg/mL)
*S. aureus*	250	15.62	15.62	≥250	0.125 *
*B. subtilis*	250	3.9	15.62	250	1
*E. coli*	125	62.5	31.25	15.62	1
*P. aeruginosa*	250	125	15.62	31.25	1
*P. mirabilis*	250	125	31.25	≥250	1
*S. typhimurium*	250	250	31.25	≥250	2

* As standard agents, penicillin G was used only for *S. aureus*, and gentamicin was used for the other bacteria.

**Table 5:** Antifungal MIC results (mg/mL) for different extracts.

Fungi	Methanol	Ethyl Acetate	Diethyl Ether	Hexane	Standard (µg/mL)
*C. albicans*	250	15.62	15.62	1.95	2 *
*C. glabrata*	125	15.62	7.81	1.95	2
*C. parapsilosis*	15.62	15.62	15.62	1.95	1
*C. krusei*	31.25	3.9	7.81	0.97	4
*P. chrysogenum*	7.81	31.25	7.81	3.9	1
*A. fumigatus*	250	15.62	7.81	0.97	1

* Fluconazole was used as a standard agent.

## Data Availability

All data used to support the findings of this study are included within the article.

## References

[1] Kenny O., Smyth T.J., Walsh D., Kelleher C.T., Hewage C.M., Brunton N.P. (2014). Investigating the potential of under-utilised plants from the *Asteraceae* family as source of natural antimicrobial and antioxidant extracts. Food Chem..

[2] Demirtas I., Tufekci A.R., Yaglioglu S., Elmastas M. (2017). Studies on the antioxidant and antiproliferative potentials of *Cirsium arvense* subsp. vestitum. J. Food Biochem..

[3] Chang N., Li Y., Zhou M., Gao J., Hou Y., Jiang M., Bai G. (2017). The hemostatic effect study of *Cirsium setosum* on regulating α1-Ars via mediating norepinephrine synthesis by enzyme catalysis. Biomed. Pharmacother..

[4] Zhao Z.W., Chang J.C., Lin L.W., Tsai F.H., Chang H.C., Wu C.R. (2018). Comparison of the hepatoprotective effects of four endemic *Cirsium* species extracts from Taiwan on CCl4-induced acute liver damage in C57BL/6 Mice. Int. J. Mol. Sci..

[5] Fernández-Martínez E., Díaz-Espinoza R., Villavicencio-Nieto M.A., Pérez-Escandón B.E., Pérez-Hernández N., Macías A., Ortíz M.I., Ponce-Monter H.A. (2007). Preliminary phytochemical and biological study of *Cirsium ehrenbergii*. Proc. West. Pharmacol. Soc..

[6] Kozyra M., Glowniak K., Los R., Mardarowicz M., Malm A., Szlapak A. (2009). GC/MS analysis of the essential oil isolated from the herb of *Cirsium vulgare* (Savi.) Ten. And its antimicrobial activity. Anna. UMCS Pharm..

[7] Ma Q., Jiang J.G., Zhang X.M., Zhu W. (2018). Identification of luteolin-7-O-beta-D-glucuronide from *Cirsium japonicum* and its anti-inflammatory mechanism. J. Funct. Foods..

[8] Strawa J., Wajs-Bonikowska A., Leszczynska K., Sciepuk M., Nazaruk J. (2016). Chemical composition and antioxidant, antibacterial activity of *Cirsium rivulare* (Jacq) All. roots. Nat. Prod. Res..

[9] Banaras S., Javaid A., Shoaib A., Ahmed E. (2017). Antifungal activity of *Cirsium arvense* extracts against phytopathogenic fungus *Macrophomin aphaseolina*. Planta Daninha.

[10] Maqbool M., Ajaib M., Ishtiaq M., Mushtaq W., Azam S., Haroon M., Azam A., Shahzaman M. (2017). Investigation of antimicrobial and antioxidant activities of *Cirsium arvensis* (L.) scop. from district Bhimber of Azad Jammu and Kashmir. J. Chem. Soc. Pak..

[11] Liao Z., Chen X., Wu M. (2010). Antidiabetic effect of flavones from *Cirsium japonicum* DC. in diabetic rats. Arch. Pharm. Res..

[12] Liu S., Zhang J., Li D., Liu W., Luo X., Zhang R., Li L., Zhao J. (2007). Anticancer activity and quantitative analysis of flavone of *Cirsium japonicum* DC. Nat. Prod. Res..

[13] Luo W., Wu B., Tang L., Li G., Chen H., Yin X. (2021). Recent research progress of *Cirsium* medicinal plants in China. J. Ethnopharmacol..

[14] Klinkhamer P.G.L., De Jong T.J. (1993). *Cirsium vulgare* (Savi) Ten.: (*Carduus lanceolatus* L., *Cirsium lanceolatum* (L.) Scop., non Hill). J. Ecol..

[15] Sabudak T., Orak H.H., Gulen D., Ozer M., Caliskan H., Bahrisefit I., Cabi E. (2017). Investigation of some antibacterial and antioxidant properties of wild *Cirsium vulgare* from Turkey. IJPER.

[16] Nazaruk J., Szok Ł. (2009). The qualitative and quantitative analysis of phenolic acids and flavonoids in *Cirsium* spp.. Herba Pol..

[17] Kozyra M., Głowniak K. (2013). Phenolic acids in extracts obtained from the flowering herbs of *Cirsium vulgare* (Savi) Ten. growing in Poland. Acta Soc. Bot. Pol..

[18] Kozyra M., Biernasiuk A., Malm A., Chowaniec M. (2015). Chemical compositions and antibacterial activity of extract sobtained from the in florescences of *Cirsium canum* (L.) all. Nat. Prod. Res..

[19] Fernández-Martínez E., Jiménez-Santana M., CentenoÁlvarez M., Torres-Valencia J.M., Shibayama M., Cariño Cortés R. (2018). Hepatoprotective Effects of Nonpolar Extracts from Inflorescences of Thistles *Cirsium vulgare* and *Cirsiu mehrenbergii* on Acute Liver Damage in Rat. Pharmacogn. Mag..

[20] Aggarwal G., Kaur G., Bhardwaj G., Mutreja V., Sohal H.S., Nayik G.A., Bhardwaj A., Sharma A. (2022). Traditional Uses, Phytochemical Composition, Pharmacological Properties, and the Biodiscovery Potential of the Genus *Cirsium*. Chemistry.

[21] Shahrajabian M.H. (2021). Spear Thistle (*Cirsium vulgare* L.) and Ramsons (*Allium ursinum* L.), impressive health benefits and high-nutrient medicinal plants. Pharmacogn. Commun..

[22] Rabi T., Bishayee A. (2009). Terpenoids and breast cancer chemoprevention. Breast Cancer Res. Treat..

[23] Miyazawa M., Yamafuji C., Ishikawa Y. (2005). Volatile Components of *Cirsium japonicum* DC. J. Essent. Oil Res..

[24] Orhan I., Deliorman-Orhan D., Ozcelik B. (2009). Antiviral activity and cytotoxixity of the lipophilic extracts of various edible plants and their fatty acids. Food Chem..

[25] Gallo M.B.C., Sarachine M.J. (2009). Biological activities of Lupeol. Int. J. Biomed. Pharmaceut. Sci..

[26] Oliveria F.A., Chaves M.H., Almeida F.R.C., Lima R.C.P., Silva R.M., Maia J.L., Brito G.A., Santos F.A., Rao V.S. (2005). Protective effect of alpha- and beta-amyrin, a triterpene mixture from *Protium heptaphyllum* (Aubl.) March. trunk wood resin, against acetamin ophen-induced liver injury in mice. J. Ethnopharm..

[27] Fatima N., Rizwan M., Hobani Y.H., Marwan A.S., Kumar B.V., Sunosi R.A., Abdulwahab S.I., Areeshi M.Y., Alvi A., Oriaby M.E. (2017). Gas Chromatography/Mass Spectrometry analysis of *Catha edulis* Forks, A psycho stimulant revealed potent solvent dependent antimicrobial activity. J. Pharmacogn. Phytochem..

[28] Iyer S., Millar T., Clemens S., Zachgo S., Giblin M., Taylor D., Sanchez L.K.J., Cerda-Olmedo E., Martinez-Force E. (1998). Advances in Plant Lipid Research.

[29] Tava A., Cunico C., Cremona R., Piccinini E. (1996). Isomeric composition of the ester fraction from epicuticular waxes of *Festuca arundinacea* Schreb. J. High. Resolut. Chromatogr..

[30] Smaoui S., Mathieu F., Fguira B.F., Lilia M., Georges M.L. (2012). Taxonomy, purification and chemical characterization of four bioactive compounds from new *Streptomyces* sp. TN256 strain. World J. Microbiol. Biotechnol..

[31] El-Sakhawy F.S., El-Tantawy M., Ross S.A., El-Sohly M.A. (1998). Composition and antimicrobial activity of the essential oil of *Murraya exotica* L.. Flavour. Fragr. J..

[32] Leventhal L.J., Boyce E.G., Zurier R.B. (1993). Treatment of rheumatoid arthritis with gammalinolenic acid. Ann. Intern. Med..

[33] Reifen R., Karlinsky A., Stark A.H., Berkovich Z., Nyska A. (2015). alpha-Linolenic acid (ALA) is an anti-inflammatory agent in inflammatory bowel disease. J. Nutr. Biochem..

[34] Aparna V., Dileep K.V., Mandal P.K., Karthe P., Sadasivan C., Haridas M. (2012). Anti-inflammatory of n-hexadecanoic acid: Structural evidence and kinetic assessment. Chem. Biol. Drug Des..

[35] Nazaruk J., Chłędzik S., Strawa J., Bazydło K., Wajs-Bonikowska A. (2017). Chemical composition and antioxidant activity of *Cirsium vulgare* (Savi) Ten. inflorescences. Nat. Pro. Commun..

[36] Griškevičienė U., Marksa M., Ževžikovienė A., Kazlauskienė D., Vainorienė R., Ževžikovas A., Ivanauskas L. (2021). *Cirsium vulgare* leaves: Isolation and identification of phenolic compounds. Chemija.

[37] Malejko J., Nalewajko-Sieliwoniuk E., Nazaruk J., Siniło J., Kojło A. (2014). Determination of the total polyphenolic content in *Cirsium palustre* (L.) leaves extracts with manganese(IV) chemiluminescence detection. Food Chem..

[38] Zhao Z.W., Chang H.C., Ching H., Lien J.C., Huang H.C., Wu C.R. (2021). Antioxidant effects and phytochemical properties of seven Taiwanese *Cirsium* species extracts. Molecules.

[39] Karasakal A., Demirci A.S., Tokatlı Demirok N., Cabi E. (2015). Antioxidant, antimicrobial activities and total flavonoid contents of *Cirsium bulgaricum* DC. leaf extracts. Marmara Pharm. J..

[40] Boga M., Yilmaz P.K., Cebe D.B., Fatima M., Siddiqui S., Kolak U. (2014). Chemical constituents and biological activities of *Cirsium leucopsis*, *C. sipyleum*, and *C. eriophorum*. Z. Naturforsch C J. Biosci..

[41] Borawska M.H., Czechowska S.K., Markiewicz R., Socha K., Nazaruk J., Pałka J., Isidorov V.A. (2010). Enhancement of antibacterial effects of extracts from *Cirsium* species using sodium picolinate and estimation of their toxicity. Nat. Prod. Res..

[42] Loizzo M.R., Statti G.A., Tundis R., Conforti F., Ando’ S., Menichini F. (2004). Antimicrobial activity and cytotoxicity of *Cirsium tenoreanum*. Fitoterapia.

[43] Nazaruk J., Jakoniuk P. (2005). Flavonoid composition and antimicrobial activity of *Cirsium rivulare* (jacq.) All. flowers. J. Ethnopharmacol..

[44] Gadisa E., Tadesse E. (2021). Antimicrobial activity of medicinal plants used for urinary tract infections in pastoralist community in Ethiopia. BMC Complement. Med. Ther..

[45] Kebede T., Gadisa E., Tufa A. (2021). Antimicrobial activities evaluation and phytochemical screening of some selected medicinal plants: A possible alternative in the treatment of multi drug-resistant microbes. PLoS ONE.

[46] Shahid M., Hussain M., Shah G.M., Rahman K.U., Ullah A., Abbasi S. (2022). Preliminary Study of the Phytochemical analysis, Antimicrobial, Antioxidant and Cytotoxic activity of *Cirsium swaticum*. J. Xi’an Shiyou Univ. Nat. Sci. Ed..

[47] Ozcelik B., Deliorman Orhan D., Karaoğlu T., Ergun F. (2005). Antimicrobial activities of various *Cirsium hypoleucum* extracts. Ann. Microbio..

[48] Zhishen J., Mengcheng T., Jianming W. (1999). The determination of flavonoid contents in mulberry and their scavenging effects on superoxide radicals. Food Chem..

[49] Re R., Pellegrini N., Proteggente A., Pannala A., Yang M., Rice-Evans C. (1999). Antioxidant activity applying an improved ABTS radical cation decolorization assay. Free Radic. Biol. Med..

[50] Benzie I.F.F., Strain J.J. (1996). The ferric reducing ability of plasma (FRAP) asameasure of “antioxidantpower”: The FRAP assay. Anal. Biochem..

[51] Miller H.E. (1971). A simplified method for evaluation of antioxidant. J. Am. Oil Chem. Soc..

[52] Apak R., Guclu K., Ozyurek M., Karademir S.E. (2004). Novel total antioxidant capacity index for dietary polyphenols and vitamins C and E, using their cupric ion reducing capability in the presence of neocuproine: CUPRAC method. J. Agric. Food Chem..

[53] Robak J., Gryglewski R.J. (1988). Flavonoids are scavengers of superoxides anions. Biochem. Pharmacol..

[54] (2008). Reference Method for Broth Dilution Antifungal Susceptibility Testing of Filamentous Fungi-Second Eddition.

[55] (2012). Reference Method for Dilution Antimicrobial Susceptibility Tests for Bacteria That Grow Aerobically. Approved Standard-NinthEdition.

[56] (2012). Reference Method for Broth Dilution Antifungal Susceptibility Testing of Yeasts; 4th Informational Supplement.

